# Stand Structure and Substrate Diversity as Two Major Drivers for Bryophyte Distribution in a Temperate Montane Ecosystem

**DOI:** 10.3389/fpls.2017.00874

**Published:** 2017-05-26

**Authors:** Yun Chen, Shuai Niu, Peikun Li, Hongru Jia, Hailiang Wang, Yongzhong Ye, Zhiliang Yuan

**Affiliations:** ^1^College of Forestry, Henan Agricultural UniversityZhengzhou, China; ^2^College of Life Sciences, Henan Agricultural UniversityZhengzhou, China; ^3^Educational Administration Department, Henan Finance and Taxation CollegeZhengzhou, China; ^4^Xiaoqinling National Nature ReserveLingbao, China

**Keywords:** bryophytes, elevation gradient, species diversity, species protection, variation partitioning

## Abstract

Elucidating the major drivers of bryophyte distribution is the first step to protecting bryophyte diversity. Topography, forest, substrates (ground, tree trunks, roots, rocks, and rotten wood), and spatial factor, which factors are the major drivers of bryophyte distribution? In this study, 53 plots were set in 400 m^2^ along the elevation gradient in Xiaoqinling, China. All bryophytes in the plots were collected and identified. Regression analysis was used to examine the relationship between bryophyte and substrate diversity. We compared the patterns of overall bryophyte diversity and diversity of bryophytes found on the ground, tree, and rock along elevational gradients. Canonical correspondence analysis was applied to relate species composition to selected environmental variables. The importance of topography, forest, substrates, and spatial factors was determined by variance partitioning. A total of 1378 bryophyte specimens were collected, and 240 species were identified. Bryophyte diversity was closely related to substrate diversity. The overall bryophyte diversity significantly increased with elevation; however, the response varied among ground, tree, and rock bryophytes. Tree diversity and herb layer were considered important environmental factors in determining bryophyte distribution. Species abundance was best explained by stand structure (17%), and species diversity was best explained by stand structure (35%) and substrate (40%). Results directly indicated that substrate diversity can improve bryophyte species diversity. The effects of micro-habitat formed by stand structure and substrate diversity were higher than those of spatial processes and topography factors on bryophyte distribution. This study proved that the determinant factors influencing bryophyte diversity reflect the trends in recent forest management, providing a real opportunity to improve forest biodiversity conservation.

## Introduction

Bryophytes are undoubtedly one of the earliest and most diversified groups of land plant, constituting an important part in vegetation diversity (Song et al., 2015). Bryophytes play important roles in the ecosystem. In particular, these species are involved in water balance and nutrient accumulation (Ódor et al., 2013). However, bryophytes are smaller than vascular plants and are often ignored in ecological studies (Stehn et al., 2010).

Elucidating the major drivers of bryophyte distribution is the firststep to conserving its diversity (Smith and Stark, 2014). Ecologists have conducted numerous explorations regarding the major drivers of vascular plant distribution. Most of the studies concluded that the coexistence of vascular plants is the joint result of niche and neutral theories (Jia et al., 2015; Chen et al., 2016). In recent years, neutral and niche theories have attracted considerable attention to explain this mechanism (Barot, 2004; Jia et al., 2015). Niche theory states that different species adapt to different habitats and are restricted by various factors (Hutchinson, 1957; Valladares et al., 2015; Conradi et al., 2017). Meanwhile, the neutral theory states that species have equal competitive abilities, and thus species patterns are only generated by dispersal limitation (Hubbell, 2001; Chen et al., 2016; Hidalgo et al., 2017). Bryophytes are reproduced by spores spreading, and dispersal limitation is one of the key processes determining the spatial structure of bryophyte distribution. Assuming that dispersal is limited in space (Hubbell, 2001), and environmental heterogeneity has been well sampled (Yuan et al., 2011), the existence of spatially structured variations of bryophyte distribution independent of the environmental fraction can indicate the importance of dispersal processes. However, the studies on the relative importance of environmental heterogeneity and spatially structured processes in explaining bryophyte distribution are relatively few. Although some ecologists conducted a few positive exploration (Mota de Oliveira et al., 2009; Silva et al., 2014; Smith and Stark, 2014), the major drivers of bryophyte distribution remain unclear.

What are the major drivers of bryophyte distribution? Smith and Stark (2014) investigated an arid desert that showed that species distribution of the bryophyte community is attributed to the two theories. Silva et al. (2014) studied the β-diversity of bryophytes on rocky outcrops and reported that randomization plays an important role in their distribution. Mota de Oliveira et al. (2009) studied the coexistence of trunk-epiphytic bryophytes and found that the role of the niche process is greater than that of the neutral process. However, these studies did not consider the roles of micro-habitats formed by stand structure and substrate diversity.

The spatial pattern of species along the elevational gradients is a basic pattern in biogeography, ecology, and conservation biology (Gaston, 2000), which also provides fundamental insights into the mechanisms of species distribution (Anderson et al., 2011). The species diversity of bryophytes along the elevation gradients has four major patterns: increasing richness with increasing elevation (Gradstein et al., 1989; Stehn et al., 2010), decreasing diversity with increasing elevation (Tusiime et al., 2007), hump-shaped distribution (Wolf, 1993; Ah-Peng et al., 2012; Song et al., 2015), or absence of any clear altitude trend (Andrew et al., 2003; Bruun et al., 2006; Sun et al., 2013). However, the growth and spread of bryophytes may be different among species that grow on different substrates, such as ground, tree, and rock bryophytes (During, 1979; Spitale, 2016). The patterns of overall bryophyte diversity may mask the true patterns of bryophyte diversity on different substrates. The study of the pattern comparison of bryophyte diversity on ground, tree, and rock is still rare.

Bryophytes inhabit a number of different substrates, such as trees, dead wood, rock, and ground in forests (Scott, 1994). The roles of substrate diversity in forest ecosystems in maintaining bryophyte diversity are frequently acknowledged (Turner and Pharo, 2005). Based on the substrate types, researchers conduct many positive explorations regarding the diversity and distribution of bryophytes (Soderstrom, 1993; Rambo and Muir, 1998; Pharo and Beattie, 2002; Szövényi et al., 2004). However, the difference of major drivers of bryophyte distribution among bryophytes on ground, tree, and rock remains unclear.

Forest management aims to harmonize timber production with biodiversity conservation to accomplish the sustainable forest management (Ódor et al., 2013). Biodiversity conservation is one of the important goals of sustainable forest management. Bryophytes are important part of biodiversity. Bryophytes can sensitively respond to environmental changes, and it is also an indicator for forest management (Nascimbene et al., 2014). The aim of this study is to improve the ecological understanding of bryophyte species distribution in forests in the context of sustainable forest management. We hope that this study can provide a reference for the protection of species diversity.

Xiaoqinling is a national nature reserve, a typical temperate deciduous broad-leaved forest. The communities have high diversity, clear layers of trees, shrubs, and herbs (Ye et al., 2004). Human disturbance is little, and bryophytes in Xiaoqinling are relatively rich (Ye et al., 2004). The highest point in the Nature Reserve is 2413.8 m, which is also the highest in the Henan province (Ye et al., 2004). So it is a natural platform for studying the factors driving the bryophyte species distribution. In this study, we set up 53 plots in 400 m^2^ along the altitudinal gradient in Xiaoqinling National Nature Reserve. We collected and identified bryophytes in all substrates within the plots. We focused on three issues: (1) How many bryophyte species exist and the difference of bryophyte diversity among different substrates; (2) What is the distribution pattern of bryophyte diversity along elevational gradients and the differences in the patterns of bryophytes among different substrates; (3) Topography, forest, substrates, and spatial factor, which factors are the major drivers of bryophyte distribution and the conservation implications for forest management in the forest ecosystem?

## Materials and Methods

### Study Area

Xiaoqinling National Nature Reserve is located in Lingbao (34° 23′–34° 31′ N, 110° 23′–110° 44′ E in Henan Province, China). The nature reserve covers 31 km from east to west and 12 km from north to south, and the total area is 15160 ha. The elevation is 308–2413.8 m, the annual average temperature is 5.9–14.0°C, the frost-free period is 170–215 days, and the annual precipitation is 506–719.2 mm (Ye et al., 2004). The most common soil types are alluvial soils and brown soil (pH of the 0–30 cm layer is 6.2–7.0) (Ye et al., 2004).

The area has rich biological resources with a diverse and high vegetation composition. Four vegetation belts exist along with elevation: scrub meadow zone (less than 1000 m), deciduous broad-leaved forest zone (1100–1700 m), mixed coniferous broad-leaved forest zone (1700–2000 m), and elfin-wood zone (more than 2000 m) (Ye et al., 2004). The forest coverage rate is 81.2%. The dominant species in forest were *Quercus variabilis Bl., Quercus aliena Bl., Quercus glandulifera* var. *brevipetiolata Nakai, Betula luminifera H. Winkl., Betula albosinensis Burk.*, and *Pteroceltis tatarinowii Maxim.*

### Sampling Design

We established 53 20 m × 20 m plots along the elevation gradient from the foothills (1020 m) to the peak of the mountain (2413 m) in August 2012. And data collected included species name of trees, number of individual trees, diameter at breast height (DBH), and height of all trees with DBH ≥ 1 cm. A ruler was used to measure the plant canopy length of east–west (EW) and north–south (NS) directions. Tree crown was calculated as *EW*(m) × *NS*(m), where *EW* is the plant canopy length of the EW direction and *NS* is the plant canopy length of the NS direction (Martens et al., 1991). A shrub community of 5 m × 5 m subplot was randomly selected within each 20 m × 20 m plot to record all shrub species, the number of individuals, and coverage. The height of each shrub species also was recorded. Four herb plots in 1 m × 1 m plot in each corner of the tree plot were also chosen, in which the data collected includes herbs species name, abundance, coverage, and height of each herb species. The longitude, latitude, and elevation of the plots were recorded by GPS. A geological compass and a clinometer were used to record aspect and slope, respectively.

We collected all bryophyte species in 53 plots of 20 m × 20 m and recorded substrate types, including ground, dead trees, rotten woods, dead leaves, trunks, roots, and rocks. All bryophyte species, including the different species and the same species at different points in the plot, were collected. We carefully checked the plot from every corner of a plot to avoid the omissions of bryophyte collections. Specimen collectors have had professional training before specimen collection. The training included basic characteristics of bryophytes, field identification of bryophytes, and field specimen collection methods. A total of 1378 specimens were collected. All specimens were identified by the microscope. Voucher specimens were deposited in the bryophyte herbarium of the Institute of Henan Agricultural University.

### Stand Structure, Topographical, and Spatial Data

A total of 12 variables, namely, tree stand density, tree basal area, tree crown, tree diversity, shrub density, shrub diversity, shrub height, shrub crown, herb density, herb diversity, herb cover, and herb height, were used to represent the stand structure. Tree stand density is the number of individual trees in a plot. Tree basal area was calculated as π × *R*^2^, where *R* is the radius at the height of 1.3 m. Tree crown is the sum of crowns in a plot. Simpson’s diversity index (Hunter and Gaston, 1988) was used to measure tree diversity. Indexes for shrub and herb were calculated using methods similar to those of a tree. Shrub and herb height are species average height. Shrub and herb density is the number of individual in a plot. Moreover, the elevation, slope, and aspect of every plot were used to represent topographical variables. Slope and aspect were converted into sinusoidal form during analysis.

Spatial variables were obtained by principal coordinates of neighbor matrices (PCNM) according to Borcard and Legendre (2002) and Legendre et al. (2009). PCNM variables represent the spatial relationship between the plots. These variables more precisely represent spatial patterns than the Euclidean distance matrix, geographic coordinates, and cubic trend surface equation (Borcard and Legendre, 2002). This method is based on the calculation of a principal coordinate analysis from a truncated matrix of Euclidean distances among plots. The PCNM characteristic function was obtained by “spacemakeR” package in R language software (Blanchet et al., 2008).

### Statistical Procedures

Regression analysis is one of the most robust methods for the relations between the variables. We examined how species diversity of all bryophytes was related to elevation using regression analysis, with significance at *p* < 0.05. Simpson’s diversity index measured species diversity. This index is one of the most commonly used methods for determining species diversity (Hunter and Gaston, 1988). We also examined the relation between species diversity of all bryophytes and substrate diversity using regression analysis. Based on the number of substrates in a plot, such as rock, ground, tree trunks, roots, and rotten wood, Simpson’s diversity index also was used to calculate the substrate diversity, so that the differences in different calculation methods can be avoided.

All bryophytes in the plots are classified into three main groups based on substrate: ground bryophytes, tree bryophytes, and rock bryophytes. The bryophytes found on the dead trees, rotten woods, dead leaves, trunks and roots are relatively few in number, and closely related to trees. So the bryophytes are classified as tree bryophytes. If there are no bryophytes in some plots, the plots will not be considered in the analysis process. Because of the substrate diversity have no method to calculate if there are no bryophytes in a plot. We examined the relation between substrate diversity and species diversity of ground bryophytes, tree bryophytes, and rock bryophytes using regression analysis. Linear regression was conducted in R 2.15.3 with Base Package (R Development Core Team).

Canonical correspondence analysis (CCA) is a simple method for arranging species along environmental variables. CCA can be used both for detecting species–environment relations and investigating specific questions regarding the response of species to environmental variables (Ter Braak, 1987). Prior to CCA analyses, detrended correspondence analysis (DCA) was conducted to detect the length of the first two axes and determine whether the linear or unimodal model should be selected. Unimodal distribution model, i.e., CCA, was selected because DCA showed a maximum axis length of more than four (Legendre and Legendre, 1998). In this study, CCA was used to determine the effects of stand structure, topographical factors, spatial factors, and substrate diversity on the bryophyte community. In CCA, species matrix is the species abundant in plots (number of individuals is more than one). Bryophytes are presence or absence at some point in one plot, abundant data of bryophytes can be obtained. The environmental matrix comprises stand structure, topographic variables, spatial variables, and substrate diversity. Stand structure includes tree stand density, tree basal area, tree crown, tree diversity, shrub density, shrub diversity, shrub height, shrub crown, herb density, herb diversity, herb cover, and herb height. Topographic variable includes elevation, slope, and aspect. Spatial variables represented by PCNM variables. Fifteen spatial PCNM variables were created in this study and used to model spatial structure at different scales. PCNM1 represents the spatial information of the entire research scale which belongs to a broad scale. PCNM2 to PCNM15 describe the fine-scale information. Stand structure, topographic variables, and spatial variables were forward selected by the “forward.se” function in R language software with “packfor” packages. Thus, a few environmental factors were removed, and the model was simplified. Moreover, Monte Carlo permutation test was used to analyze whether the model reached a significant level (*P* < 0.05). The “envfit” function in the R language software with “vegan” package (Oksanen et al., 2007) was used to test the significance of each environmental factor and bryophyte species distribution. The same methodology was used to determine the effects of stand structure, topographical factors, and spatial factors on the ground bryophytes, tree bryophytes, and rock bryophytes, respectively. PCNM variables were created by “PCNM” packages (Blanchet et al., 2008). CCA and DCA analyses were conducted in R 2.15 with “vegan” package (Oksanen et al., 2007) (R Development Core Team^1^).

Variance partitioning is useful in evaluating the major drivers of different species in community ecology (Violle et al., 2012; Coyle, 2017). Variation partitioning was performed to divide the variation in bryophytes species distribution and diversity, respectively, and explore the influence of spatial processes and topography factors, stand structure, and substrate diversity on bryophyte species distribution and species diversity. The dependent variable in variance partitioning is bryophyte species abundance matrix, which was transformed by “hellinger” (Legendre and Gallagher, 2001), or species diversity, which was calculated by Simpson’s diversity index. Hellinger transformations are appropriate alternatives giving low weights to rare species (Legendre and Gallagher, 2001). And this transformation might provide better resolutions for species by making them behave more like a Gaussian distribution (Griffith and Peres-Neto, 2006). Variance partitioning was also used to determine the effects of stand structure, topographical factors, and spatial factors on the ground bryophytes, tree bryophytes, and rock bryophytes, respectively. Variance partitioning was computed using the “vegan” library (Oksanen et al., 2007) of the R statistical language.

## Results

### Bryophyte Diversity in Xiaoqinling

Based on the 1378 bryophyte specimens collected in 53 plots, a total of 240 bryophyte species were collected, which belong to 33 families and 89 genera (Supplementary Table S1). Bryophyte abundance in 53 plots is provided in Supplementary Table S2. A total of 126 trees, 130 shrubs, and 263 herbs species were recorded. Statistics of the bryophyte diversity and stand structure are provided in **Table 1**. The bryophyte diversity (0.89) is second only to herbs diversity (0.92) and is far higher than the diversity of trees (0.75) and shrubs (0.77) (**Table 1**). Therefore, bryophytes have important significance for species diversity in this region.

**Table 1:** Overview of bryophytes, trees, shrubs, and herbaceous plants in 53 plots.

Variables	Minimum	Average value (±*SD*)	Maximum
Bryophyte diversity	0.77	0.89 ± 0.04	0.95
Ground bryophyte diversity	0.50	0.76 ± 0.12	0.92
Tree bryophyte diversity	0.50	0.77 ± 0.14	0.95
Rock bryophyte diversity	0.50	0.83 ± 0.10	0.94
Substrate diversity	0.00	0.48 ± 0.19	0.81
Tree diversity	0.02	0.75 ± 0.12	0.90
Tree stand density	20.00	62.20 ± 20.97	129.00
Tree basal area (m^2^)	0.10	1.10 ± 0.17	4.80
Tree crown (m^2^)	72.00	213.30 ± 62.03	385.50
Shrub diversity	0.46	0.77 ± 0.12	0.92
Shrub density	8.00	25.80 ± 12.26	56.00
Shrub height (cm)	133.50	157.21 ± 45.72	247.68
Shrub crown (m^2^)	5.70	18.90 ± 8.98	43.00
Herb diversity	0.81	0.92 ± 0.15	0.96
Herb density	59.00	281.10 ± 156.93	626.00
Herb height (cm)	6.15	10.45 ± 3.04	22.90
Herb cover (m^2^)	2.71	5.494 ± 0.86	8.78

*Diversity was measured by Simpson’s diversity index.*

**FIGURE 1:**
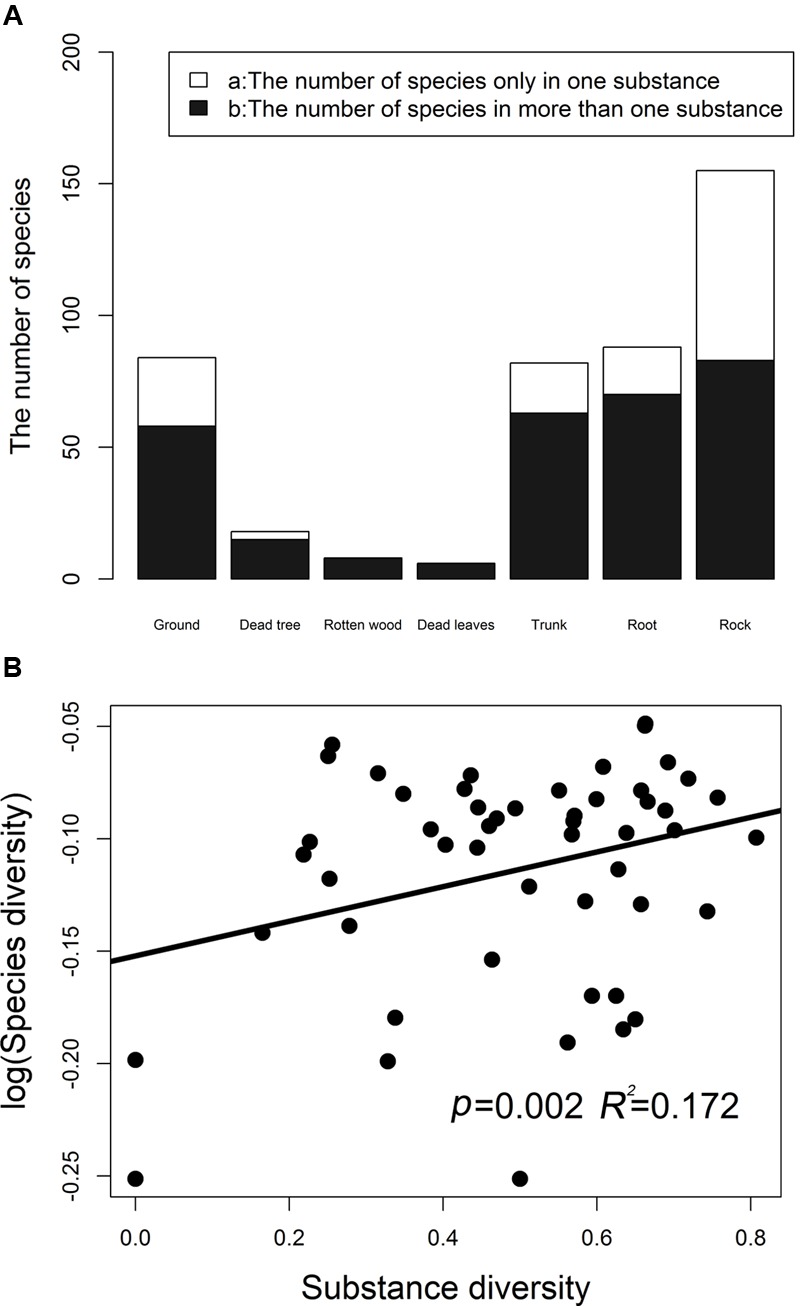
The number of bryophyte species in different substrates **(A)**. **(B)** Shows the relationship between the bryophytes species diversity (log-transformed) and substrate diversity. Simpson’s diversity index was used to measure species diversity and substrate diversity, respectively. Substrate diversity was calculated based on the number of substrates in a plot.

Bryophyte diversities remarkably differed among different substrates, and 57.5% species tended to be distributed in one kind of substrate. The numbers of bryophyte species in different substrate types are illustrated in **Figure 1A**. Species of saxicolous bryophytes are the richest species. A total of 72, 26, 19, 18, and 3 bryophyte species were recorded in the rock, ground, tree trunks, roots, and dead trees, respectively. Rock bryophytes are the richest. The regression analysis indicated that the overall bryophyte diversity significantly increased with the substrate diversity (**Figure 1B**). For overall bryophyte diversity, substrate diversity explained 17.20% of the total variance (*p* = 0.0020).

### Elevation Patterns of Species Diversity

**FIGURE 2:**
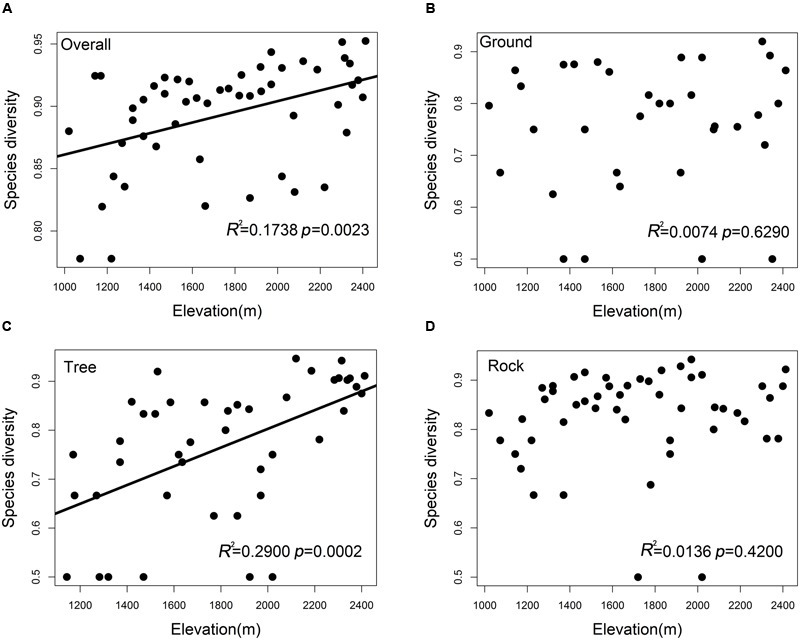
Species diversity patterns of **(A)** overall bryophytes, **(B)** ground bryophytes, **(C)** tree bryophytes, and **(D)** rock bryophytes along the elevation. Simpson’s diversity index was used to measure species diversity.

The overall bryophyte diversity significantly increased with the elevation (*R*^2^ = 0.1738, *p* = 0.0023); however, bryophyte diversity varied among ground, tree, and rock bryophytes (**Figures 2A–D**). The tree bryophyte diversity exhibited highly similar trends along the elevation gradient with the overall bryophyte diversity (*R*^2^ = 0.2900, *p* = 0.0002). The diversity of ground (*p* = 0.6290) and rock bryophytes (*p* = 0.4200) did not have a significant correlation to elevation.

### Influence of Environmental Factors on Bryophyte Communities

**FIGURE 3:**
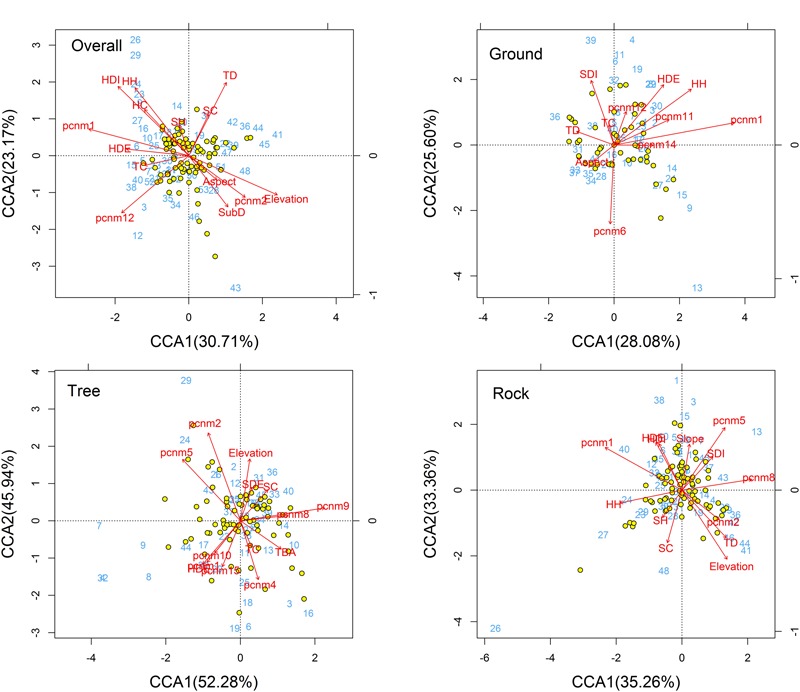
CCA ordination diagram of the bryophytes data. Yellow dots and blue numbers represent species and plots, respectively. Arrows represent environmental factors. The longer the arrows represent the correlation is larger or smaller. We used the PCNM approach to introduce space as an explanatory variable. The analysis was conducted using bryophyte species abundant. Numbers on the CCA1 or CCA2 is the eigenvalue value. Abbreviations: TSD, tree stand density; TBA, tree basal area; TC, trees crown; TD, tree diversity; SDE, shrub density; SDI, shrub diversity; SH, shrub height; SC, shrub crown; HDE, herb density; HDI, herb diversity; HH, herb height; HC, herb cover; SubD, substrate diversity; pcnm, spatial PCNM variables.

The CCA ordination of all plots and bryophyte species indicated that variation in the species composition of bryophytes was mainly explained by the stand structure (Trees crown, herb diversity, herb density, and herb height) and partly by spatial factors (PCNM1, PCNM2, and PCNM12) and elevation (**Figure 3**). In the ordination diagram, environmental variables explained 30.93% of the variation in the structure of the assemblages. The first CCA axis (eigenvalue 0.30714) was positively correlated with elevation and negatively correlated with large-scale spatial patterns (PCNM1) and herb density. The second CCA axis (eigenvalue 0.23170) represented a substrate diversity gradient. In the ordination analysis, the significant variables in the permutation test were elevation, trees crown, herb density, herb diversity, herb height, PCNM1, PCNM2, and PCNM12 (**Table 2**); however, elevation, trees crown, herb diversity, and height are the most important, whereas the effects of aspect and shrub height were less influential.

**Table 2:** Significant explanatory variables in the canonical correspondence analysis (CCA).

Variables	CCA1	CCA2	*R*^2^	*P-*value
**Overall bryophytes**				
PCNM1	0.98917	-0.14675	0.4809	0.001^∗∗∗^
Elevation	-0.87080	0.49163	0.4792	0.001^∗∗∗^
TC	0.47912	0.87775	0.3361	0.001^∗∗∗^
HDI	0.82745	-0.56153	0.3277	0.002^∗∗^
PCNM12	0.64381	0.76518	0.3021	0.006^∗∗^
PCNM2	-0.70846	0.70575	0.2720	0.005^∗∗^
HDE	0.84885	-0.52864	0.1684	0.047^∗^
HH	0.96814	-0.25042	0.1562	0.050^∗^
**Ground bryophytes**				
PCNM1	0.925086	0.379758	0.6235	0.001^∗∗∗^
HH	0.993430	0.114441	0.3600	0.003^∗∗^
PCNM6	-0.587155	0.809474	0.2701	0.024^∗^
HDE	0.974585	-0.224017	0.2410	0.025^∗^
PCNM11	0.993183	0.116563	0.1962	0.047^∗^
SDI	0.095955	-0.995386	0.1917	0.044^∗^
**Tree bryophytes**				
PCNM2	0.362650	-0.931926	0.3215	0.002^∗∗^
PCNM5	0.713888	-0.700260	0.2803	0.007^∗∗^
PCNM9	-0.989549	-0.144196	0.2720	0.007^∗∗^
Elevation	-0.135150	-0.990825	0.1503	0.048^∗^
PCNM4	-0.339780	0.940505	0.1447	0.046^∗^
**Rock bryophytes**				
PCNM1	0.790720	0.612178	0.3110	0.004^∗∗^
Elevation	-0.398264	-0.917271	0.2843	0.002^∗∗^
PCNM5	-0.670222	0.742161	0.2716	0.003^∗∗^
PCNM8	-0.999660	0.026059	0.1926	0.017^∗^
HH	0.991905	-0.126984	0.1924	0.036^∗^
TD	-0.529105	-0.848556	0.1748	0.036^∗^
HDE	0.439159	0.898409	0.1208	0.045^∗^

*The variable abbreviations are the same as in **Figure 3**. ^∗^*P* < 0.05; ^∗∗^*P* < 0.01; ^∗∗∗^*P* < 0.001.*

For ground, tree, and rock bryophytes, environmental variables explained 35, 35, and 27% of the variation in the structure of the bryophytes assemblages, respectively. The CCA ordination indicated that spatial factors are the most important for ground, tree, and rock bryophytes. Meanwhile, the effects of elevation are also important factors for tree bryophytes, and the effects of shrub and herb are more important factors for ground and rock bryophytes, respectively (**Figure 3**).

### The Major Drivers of Bryophyte Distribution

**FIGURE 4:**
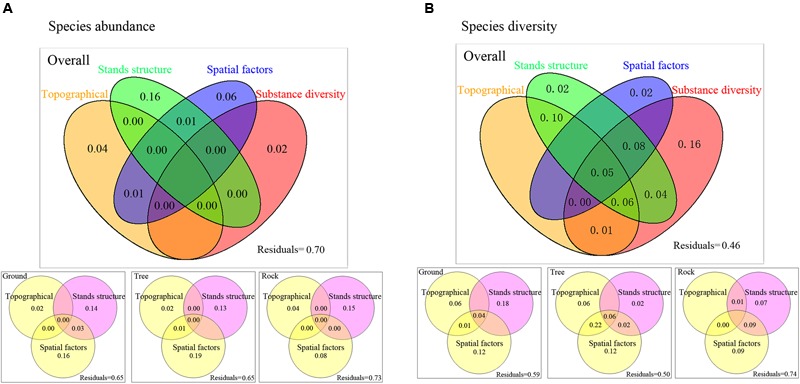
Variance partitioning for the effects of topographical factors, spatial factors, stands structure, and substrate diversity on bryophyte species abundance **(A)** and species diversity **(B)**. Values less than zero are not shown. Abundant data was calculated based on the presence or absence of bryophyte species at some point in one plot. Species diversity was measured by Simpson’s diversity index. Spatial variables represented by PCNM variables.

For species abundance, variation partitioning results show that topographical factors, stand structure, spatial factors, and substrate diversity explained 30.93% of the variation in the overall bryophyte distribution. Of this, 16% was attributed to pure stand structure, 6% was attributed to pure spatial factors, 4% was attributed to pure topographical variables, and 2% was attributed to pure substrate diversity. The effect of stand structure was relatively remarkable (**Figure 4A**). For ground and tree bryophytes, the effects of pure spatial factors on bryophyte distribution (16 and 19%, respectively) were slightly higher than those of pure stand structure (14 and 13%), and the effects of pure topographical factors on bryophyte distribution were the lowest (2 and 2%, respectively). For rock bryophytes, the effects of pure stand structure on bryophyte distribution were the highest (15%).

For species diversity, variation partitioning results show that topographical factors, stand structure, spatial factors, and substrate diversity explained 54% of the variation in the overall bryophyte diversity. Of this, 16% was solely attributed to substrate diversity, 10% was the joint effect of topographic and stand structure, and 8% was the joint effect of topography, spatial factors, and substrate diversity (**Figure 4B**). For ground bryophytes, the effects of pure stand structure on bryophyte diversity (18%) were higher than those of pure spatial factors (12%). For tree bryophytes, the effects of pure stand structure on bryophyte diversity were the lowest (2%). For rock bryophytes, the effects of pure spatial factors on bryophyte diversity (9%) were higher than those of pure stand structure (7%).

## Discussion

### Substrate Diversity

Our results indicated that substrate diversity can improve bryophyte species diversity. The diversity of bryophytes in forest ecosystems is related to forest management practices (Hofmeister et al., 2015). As forest management leads to insufficiency of old-growth forest attributes, bryophyte diversity has declined in most managed forests (Söderström, 1988; Friedel et al., 2006). However, Lindenmayer and Laurance (2012) argued that the effects of management practices are spatially and temporarily restricted. Hofmeister et al. (2015) studied European forests and found that the bryophyte species richness in managed forests was less than in unmanaged forests. Moreover, their study showed that only unmanaged forest stands that provided suitable substrates were able to support rare bryophyte species. Hofmeister et al. (2015) indirectly showed the importance of maintaining the substrate diversity by comparing the managed and unmanaged forests.

### Species Spatial Patterns along the Elevation Gradient

Temperature, precipitation, and air humidity remarkably differ along elevation gradients (Lieberman et al., 1996). Our results showed that bryophyte diversity exhibited an increasing richness pattern with increasing elevation. Our results are also consistent with those of Gradstein et al. (1989) and Stehn et al. (2010). This is probably because bryophyte diversity increases with elevation as a result of increased moisture availability (Lee and Roi, 1979) and the ability of bryophytes to tolerate extreme conditions (Bruun et al., 2006) and partly because the degree of human disturbance is small at high elevation.

Our results show that ground, tree, and rock bryophytes exhibited different elevation patterns. The study of Söderström (1988) showed that many bryophyte species are therefore absent in forests with an uneven supply of substrate. If dispersal is limited, bryophytes will not, or rarely, appear (Söderström, 1988). Tree bryophyte diversity exhibited an increasing richness pattern with increasing elevation. This may because tree bryophytes mainly grow on the trunk and was closely related to the trees (Benítez et al., 2015). Trees are the main part of the forest ecosystem and are continuously distributed with increasing elevation. Accordingly, the growth and spread of tree bryophytes provide continuous space. However, the rocks are randomly distributed with increasing elevation, and dispersal limitation perhaps influence the spread of the bryophyte species. Therefore, the diversity of rock bryophytes did not have a significant correlation to elevation. For ground bryophytes, although it has a continuous space with species spread, it is strongly influenced by environmental spatial heterogeneity. For example, a few bryophytes prefer more shaded condition and high air humidity (Hosokawa and Odani, 1957; Lesica et al., 1991; Humphrey et al., 2002; Ranius et al., 2008), whereas some bryophytes prefer more open conditions as forest edges (Moe and Botnen, 2000; Vanderpoorten et al., 2004). Hence, the routes of ground bryophytes spread may are still restricted. Therefore, the diversity of ground bryophytes did not have a significant correlation to elevation. In this study, we discussed the distribution pattern of bryophyte only from the angle of the substrate. Other possible factors, such as the spread of bryophyte spores, were not considered here.

### Bryophyte Composition and Environmental Variables

The CCA ordination of all bryophytes indicated that variation in the species composition of bryophytes was mainly explained by the stand structure, including the diversity and crown of the tree as well as the coverage, height, density, and diversity of the herb layer. The diversity of trees can provide a diverse habitat for bryophytes (Rosabal et al., 2013), and previous research has shown that tree diversity has a close relation with bryophyte diversity (Pharo et al., 1999, 2000; Chiarucci et al., 2007). Coverage, height, density, and diversity of the herb layer could elevate local air humidity (Aude and Poulsen, 2000; Brunialti et al., 2010). Therefore, tree diversity and herb layer were considered important environmental factors in determining the bryophyte distribution. Although the diversity and crown of the tree are important factors for overall bryophytes, our results show that no tree structure factors significantly affect tree bryophyte distribution in this region. Chiarucci et al. (2007) concluded their study on vascular plants and bryophytes which have similar results with us. The relations between tree structure factors and tree bryophyte diversity needs to study and investigate further.

Furthermore, the CCA ordination indicated that spatial variables on different scales significantly affected the distribution of overall bryophytes, signifying that local dispersal ability is a key determinant of the bryophyte community structure. These findings agree with the observations of local dispersal limitation in deserts (Smith and Stark, 2014), bogs (Andersen et al., 2011), forests (Löbel et al., 2006), and land (Chen et al., 2015) bryophyte communities. However, spatial variables measured are notably not equal to the dispersal capabilities of species. Many scholars have conducted active exploration in the dispersal capabilities of bryophyte (Miles and Longton, 1992; Nathan and Muller-Landau, 2000). This will help us further understand the effects of the spatial process on bryophyte distribution.

### Community Assembly

Our results indicated that the effects of stand structure and substrate diversity on bryophyte distribution were higher than those of spatial processes and topography factors. Stand structure exhibited high explanatory power because trees, shrubs, and herbs constitute different micro-habitats (Mežaka et al., 2012). Smith and Stark (2014) showed that species distribution in the bryophyte community is attributed to the joint result of niche and neutral theories. Mota de Oliveira et al. (2009) studied the coexistence of trunk-epiphytic bryophytes and showed that the role of the niche process is greater than that of the neutral process. Our research is based on the niche and neutral theories, and we also considered the stand structure and substrate diversity. Our results are also inconsistent with those of Mota de Oliveira et al. (2009) and Smith and Stark (2014). Our results showed that the effects of micro-habitat formed by stand structure and substrate diversity were higher than those of spatial processes and topography factors on bryophyte community assembly. Vitt et al. (1995) studied the relative importance of the micro-habitat and climate in the Peatlands of Continental Western Canada, which showed that bryophyte richness is most highly correlated with habitat heterogeneity whereas climatic factors are insignificant. Thus, our results are consistent with those of Vitt et al. (1995). Micro-habitat formed by stand structure and substrate diversity are important factors for bryophyte species distribution.

When bryophytes in plots were classified into three main groups, variation partitioning results showed that the effects of spatial variables had increased. So stand structure and spatial processes are all important factors for ground, tree, and rock bryophytes. The mechanisms of community assembly may differ among ground, tree, and rock bryophytes (Halpern et al., 2014; Spitale, 2017). Our results showed that the effects of spatial processes on tree and ground bryophytes community were higher than rock bryophytes community. This may because the distribution of rock is more random and uneven. And trees are continuously distributed with increasing elevation. The distribution of tree bryophytes is affected by the spatially structured variations of tree community. Ground bryophytes also have a continuous space with species spread although it is influenced by environmental spatial heterogeneity. Therefore, the effects of spatial processes on tree and ground bryophytes community were higher than rock bryophytes community.

The study of Chang et al. (2013) showed that better environmental data could reverse the conclusions regarding community assembly processes. Substrate availability (Mills and Macdonald, 2005), climate and soil factors (Heilmann-Clausen et al., 2014), and the height of the host tree (Oliveira and Steege, 2015), as the important environmental factors for bryophyte distribution, were not considered here. Therefore, more environmental factors must be considered in future studies.

### Implications for Species Conservation

In the perspective of sustainable forest management, this study proved that the determinant factors influencing bryophyte diversity reflect on trends in recent forest management (trees crown and diversity, coverage, height, density and diversity of herb layer, and substrate diversity), providing a real opportunity to improve forest biodiversity conservation. Based on our results, the main strategy of management focusing on diversity conservation should include the following: (1) maintenance of tree species diversity. Because of the diversity of trees can provide a diverse habitat for bryophytes. (2) Presence of herb and regeneration layer. This can elevate local air humidity and suitable for bryophyte growth. (3) The maintenance of substrate diversity, rock, and dead wood in the forest must be retained. More substrates can provide continuous and diversified spaces for bryophyte growth, and reduce the dispersal limitation. (4) Ground, tree, and rock bryophytes perhaps should receive different protection strategies. Because of the major drivers of bryophytes distribution have some difference among ground, tree, and rock bryophytes. (5) Protection of bryophyte species diversity may require propagule supplementation for individual species when dispersal limitation exists.

## Author Contributions

ZY originally formulated the idea, YC developed methodology, SN, PL, HJ, HW, and YY conducted fieldwork, YC performed statistical analyses and wrote the manuscript.

## Conflict of Interest Statement

The authors declare that the research was conducted in the absence of any commercial or financial relationships that could be construed as a potential conflict of interest.

## Footnotes

1 http://www.Rproject.org
