# Supplementary material for: Stand Structure and Substrate Diversity as Two Major Drivers for Bryophyte Distribution in a Temperate Montane Ecosystem
Source: Front Plant Sci. 2017 May 26;8:874. doi: 10.3389/fpls.2017.00874 (PMC5445162; doi:10.3389/fpls.2017.00874)
Supplement: Supplementary file 1 [file Table_1.PDF]

Table S1 Bryophyte species and substrate types were recorded in 53 plots in Xiaoqinling National Nature Reserve.

Species	Substrate types		
Amblystegium serpens	Ground		
Anacamptodon fortunei mitt			Rocks
Andmodon minor var. *integerrimus*	Ground	Trees	Rocks
Andmodon minor var. *minor*		Trees	Rocks
Anoectangium aestivum			Rocks
Anoectangium clarum			Rocks
Anoectangium stracheyanum			Rocks
Anomobryum filiforme Solms	Ground		Rocks
Anomodon dentatus		Trees	Rocks
Anomodon perlingulatus			Rocks
Anomodon rotundatus			Rocks
Anomodon viticulosus		Trees	Rocks
Archilegeunea planiuscula		Trees	Rocks
Atrichum angustatum	Ground	Trees	
Atrichum henryi	Ground	Trees	Rocks
Atrichum rhystophyllum		Trees	
Atrichum var. *gracilisetum besch*		Trees	Rocks
Atrichum yakushimense			Rocks
Barbula subcontorta	Ground		
Barbula unguiculata	Ground		
Boulaya mittenii		Trees	
Brabhythecium piumosum		Trees	Rocks
Brachymenium nepalense	Ground	Trees	
Brachythecium	Ground		Rocks
Brachythecium albicans	Ground	Trees	Rocks
Brachythecium amnicola	Ground	Trees	Rocks
Brachythecium campylothallum	Ground	Trees	Rocks
Brachythecium coreanum	Ground	Trees	Rocks
Brachythecium curtum	Ground	Trees	Rocks
Brachythecium dicranoides	Ground		Rocks
Brachythecium garovaglioides	Ground	Trees	Rocks
Brachythecium glauculum	Ground		
Brachythecium moriense		Trees	Rocks
Brachythecium piligerum	Ground	Trees	Rocks
Brachythecium plumosum			Rocks
Brachythecium populeum	Ground	Trees	Rocks
Brachythecium procumbens	Ground		Rocks
Brachythecium propinnatum			Rocks
Brachythecium pulchellum	Ground	Trees	
Brachythecium reflexum	Ground		
Brachythecium salebrosum	Ground	Trees	

Brachythecium thraustum		Trees	Rocks
Brachythecium uncinifolium	Ground	Trees	Rocks
Brotherus leana	Ground		
Bryhnia brachycladula	Ground		Rocks
Bryhnia hultenii	Ground		
Bryhnia nocae-angliae	Ground	Trees	Rocks
Bryhnia trichomitria	Ground		
Bryoerythrophyllum alpigenum			Rocks
Bryum argenteum		Trees	
Bryum bornholmense	Ground		
Bryum caespiticium			Rocks
Bryum pseudotriquetrum			Rocks
Bryum radiculosum	Ground		
Bryum tuberosum			Rocks
Camptothecium		Trees	
Campylium hispidulum		Trees	
Campylium prophyriticum			Rocks
Campylopus fragilis		Trees	Rocks
Cheiloeunea chenii			Rocks
Chiloscyphus horikawana	Ground		
Chiloscyphus minor		Trees	
Chiloscyphus sinensis		Trees	
Claopodium assurgens	Ground		
Claopodium gracillimum			Rocks
Claopodium pellucinerve		Trees	
Claopodium rugulosifolium		Trees	
Conocephalum conicum	Ground		Rocks
Ctenidium hastile			Rocks
Cyrto-hypnum gratum		Trees	
Cyrtohypnum pygmaeum	Ground	Trees	Rocks
Cyto-hypnum fuscatum	Ground	Trees	
Cyto-hypnum versicolor			Rocks
Cyto-hypnum vestitissimum			Rocks
Desmatodon latifolius			Rocks
Didymodon rigidulus		Trees	
Drepanocladus revolvens		Trees	
Ectropothecium buitenzorgii			Rocks
Encalypta apathulata			Rocks
Entodon acutifolius		Trees	Rocks
Entodon aeruginosus	Ground		
Entodon dolichocucullatus	Ground	Trees	Rocks
Entodon longifolius		Trees	Rocks
Entodon luridus		Trees	
Entodon macropodus		Trees	

Entodon obtusatus	Ground	Trees	Rocks
Entodon okamurae Broth	Ground		
Entodon plicatus	Ground		
Entodon prorepens	Ground	Trees	Rocks
Entodon pulchellus	Ground	Trees	Rocks
Entodon viridulus			Rocks
Entodontopsis wightii			Rocks
Eurbynchuium arbuscula		Trees	
Eurbynchuium filiforme	Ground	Trees	
Eurbynchuium laxirete	Ground	Trees	
Eurbynchuium longirameum	Ground	Trees	Rocks
Eurhynchium angustirete	Ground		
Eurhynchium coarctum		Trees	
Eurhynchium eu?stegium	Ground	Trees	
Eurhynchium hians			Rocks
Eurhynchium kirishimense	Ground		Rocks
Eurhynchium kirishimense Takaki	Ground		
Eurhynchium savatieri Schimp	Ground		Rocks
Eurhynchium squarrifolium	Ground		Rocks
Eurohypnum leptothallum		Trees	Rocks
Eurohypnum leptothollum	Ground		
Fabronia ciliaris	Ground	Trees	
Fissidens anomalus	Ground		Rocks
Fissidens areolatus			Rocks
Fissidens bryoides	Ground	Trees	Rocks
Fissidens cristatus			Rocks
Fissidens nobilis	Ground		
Fissidens perdecurrens			Rocks
Fissidens plagiochloides			Rocks
Fissidens strictulus			Rocks
Fissidens tosaensis			Rocks
Fissideus bryoides var. *bryouides*	Ground	Trees	Rocks
Frullania chenii			Rocks
Frullania inflexa		Trees	
Frullania polyptera		Trees	Rocks
Gollania neckerella	Ground		Rocks
Gollania robusta	Ground	Trees	Rocks
Gollania ruginosa			Rocks
Gollania sinensis		Trees	
Gollania varians	Ground		
Haplocladium angustifolium		Trees	Rocks
Haplocladium larminatii		Trees	
Haplocladium microphyllum			Rocks
Haplocladium strictulum		Trees	Rocks

Haplohymenium Flagelliforme		Trees	
Haplohymenium triste		Trees	
Herpetineuron toccoae			Rocks
Homaliodendron exiguum	Ground	Trees	Rocks
Hygroamblystegium tenax		Trees	
Hyophila anomala			Rocks
Hyophila propagulifera			Rocks
Hyophila stenophylla		Trees	Rocks
HyophilaBrid javanica		Trees	Rocks
Hypnum hamulosum		Trees	
Hypnum shensianum			Rocks
Hypnum subimponens			Rocks
Isopterygiopsis muelleriana	Ground	Trees	
Isopterygium albescens		Trees	
Isopterygium bancanum		Trees	
Isopterygium serrulatum		Trees	
Jungermannia (S.) pseudocyclops Inoue	Ground	Trees	Rocks
Jungermannia breviperianthia		Trees	
Jungermannia leiantha	Ground	Trees	Rocks
Lejeunea cavifolia	Ground	Trees	Rocks
Lejeunea sordida		Trees	
Leptobryum pyriforme	Ground		Rocks
Lescuraea radicosa			Rocks
Leskea gracilescens	Ground	Trees	Rocks
Leskea polycarpa		Trees	Rocks
Leskea scabrinervis	Ground	Trees	Rocks
Leskeella nervosa			Rocks
Lindbergia brachyptera		Trees	
Lindbergia serrulatus	Ground		
Lindbergia sinensis		Trees	Rocks
Merceyopsis sikkimensis		Trees	Rocks
Miyabea frutcella		Trees	
Mnium heterophyllum		Trees	
Mnium hornum	Ground	Trees	Rocks
Mnium laevinerve	Ground	Trees	Rocks
Mnium lycopodioides			Rocks
Mnium marginatum		Trees	
Mnium spinosum	Ground		
Oxystegus cuspidatus	Ground	Trees	Rocks
Plagiobrym giraldii			Rocks
Plagiomnium acutum	Ground		
Plagiomnium arbusculum		Trees	
Plagiomnium conferteidens	Ground	Trees	Rocks
Plagiomnium cuspidatum	Ground	Trees	Rocks

Plagiomnium integrum	Ground	Trees	Rocks
Plagiomnium maximovicgii	Ground		
Plagiomnium rhynchophorum		Trees	
Plagiomnium succulentum	Ground	Trees	Rocks
Plagiomnium tezukae	Ground		Rocks
Plagiomnium vesicatum	Ground	Trees	Rocks
Plagiothecium cavifolium	Ground		
Plagiothecium formosicum	Ground		
Plagiothecium nemorale	Ground	Trees	Rocks
Platydictya jungermannioides		Trees	
Platydictya subtilis			Rocks
Pleuroweisia schliephackei			Rocks
Pohlia cruda			Rocks
Pohlia nutans			Rocks
Pottia truncata			Rocks
Pseudosymblepharis duriuscula	Ground		
Pseudotaxiphyllum pohliaecarpum		Trees	Rocks
Ptychomitrium dentatum			Rocks
Ptychomitrium fauriei			Rocks
Ptychomitrium polyphyllum			Rocks
Ptychomitrium wilsonii			Rocks
Pylaisiadelpha yokohamae	Ground	Trees	Rocks
Pylaisiella brotheri		Trees	
Pylaisiella robusta			Rocks
Pylaisiella sewynii		Trees	Rocks
Pylaisiellapolyantha		Trees	
Pyrrhobryum latifolium			Rocks
Rhizomnium horikawae			Rocks
Rhizomnium punctatum		Trees	Rocks
Rhizomnium tuomikoskii			Rocks
Rhodobryum giganteum			Rocks
Rhodobryum ontariense		Trees	Rocks
Rhynchostegiella japonica	Ground	Trees	Rocks
Rhynchostegium fauriei			Rocks
Rhynchostegium inclinatum		Trees	
Rhynchostegium riparioides	Ground	Trees	
Rhynchostegium serpenticaule			Rocks
Rhynchostegium subspeciosum	Ground		
Schwetschkea sinica	Ground	Trees	
Schwetschkeopsis fabronia	Ground	Trees	Rocks
Sematophyllum phoeniceum		Trees	
Targionia hypophylla			Rocks
Taxiphyllum cuspidifolium			Rocks
Taxiphyllum giraldii	Ground		Rocks

Taxiphyllum taxirameum	Ground	Trees	Rocks
Teachyphyllum inflexum	Ground	Trees	Rocks
Thamnobryum subseriatum			Rocks
Thuidium cymbifolium	Ground	Trees	Rocks
Thuidium delicatulum	Ground	Trees	
Thuidium kanedae	Ground	Trees	Rocks
Thuidium philibertii			Rocks
Thuidium submicropteris			Rocks
Timmiella anomala			Rocks
Tortula princeps			Rocks
Tortula subulata		Trees	Rocks
Trachycystis microphylla		Trees	
Trachycystis ussuriensis	Ground	Trees	Rocks
Trichostomum brachydontium			Rocks
TrichostomumBruch brachydontiumBruch		Trees	Rocks
Vesicularia ferriei			Rocks
Vesicularia flaccida		Trees	Rocks
Vesicularia reticulata	Ground	Trees	Rocks
Weisia controversa		Trees	Rocks
Weisia exserta		Trees	
WeisiaHedw controversaHedw			Rocks
Weisiopsis anomala	Ground		Rocks
Wettsteinia inversa	Ground		Rocks

Table S2 Bryophyte abundance in 53 plots in Xiaoqinling National Nature Reserve. Substrate diversity was calculated based on the number of substrates in a plot.

Plot NO.	Bryophyte abundance	Liverwort abundance	Moss abundance	Substrate diversity
1	12	1	11	0.844291
2	8	0	8	0.700000
3	14	1	13	0.875000
4	14	1	13	0.880000
5	9	0	9	0.806400
6	5	0	5	0.777778
7	7	0	7	0.777778
8	13	0	13	0.829932
9	10	0	10	0.792899
10	16	0	16	0.868056
11	12	1	11	0.877551
12	16	2	14	0.880658
13	9	1	8	0.722222
14	20	0	20	0.884944
15	12	0	12	0.814815
16	20	0	20	0.886667
17	14	0	14	0.885813
18	12	0	12	0.875000
19	22	0	22	0.911844
20	14	0	14	0.896694
21	17	1	16	0.915713
22	13	1	12	0.888889
23	17	1	16	0.827160
24	7	0	7	0.790123
25	14	2	12	0.875283
26	2	0	2	0.500000
27	24	0	24	0.879501
28	16	0	16	0.858025
29	5	0	5	0.562500
30	21	1	20	0.882396
31	19	1	18	0.902778
32	14	1	13	0.899811
33	7	0	7	0.800000
34	29	2	27	0.920395
35	15	2	13	0.869806
36	19	1	18	0.893333
37	26	2	24	0.938856
38	7	0	7	0.816327
39	20	1	19	0.916955
40	10	0	10	0.777778

41	25	2	23	0.823342
42	23	1	22	0.917500
43	21	0	21	0.899691
44	9	1	8	0.819945
45	13	2	11	0.793388
46	26	4	22	0.929012
47	26	2	24	0.919184
48	11	0	11	0.853333
49	26	1	25	0.905413
50	17	2	15	0.899811
51	18	0	18	0.896030
52	21	1	20	0.878906
53	30	4	26	0.920000
